# Therapeutic vascular growth in the heart

**DOI:** 10.1530/VB-19-0006

**Published:** 2019-03-28

**Authors:** Ebba Brakenhielm, Vincent Richard

**Affiliations:** 1 Normandy University, UniRouen, Inserm (Institut National de la Santé et de la Recherche Médicale) UMR1096 (EnVI Laboratory), FHU REMOD-VHF, Rouen, France

**Keywords:** heart failure, myocardial infarction, angiogenesis, endothelial dysfunction, ischemia, perfusion, lymphangiogenesis, growth factor combinations, biopolymers, gene therapy, exosomes

## Abstract

Despite tremendous efforts in preclinical research over the last decades, the clinical translation of therapeutic angiogenesis to grow stable and functional blood vessels in patients with ischemic diseases continues to prove challenging. In this mini review, we briefly present the current main approaches applied to improve pro-angiogenic therapies. Specific examples from research on therapeutic cardiac angiogenesis and arteriogenesis will be discussed, and finally some suggestions for future therapeutic developments will be presented.

## Introduction

Over the last 15 years, the use of anti-angiogenic agents to inhibit blood vessel growth has clearly established its ‘raison d’être’ in the treatment of human diseases, including multiple types of cancer, but also vascular retinopathies (1). In sharp contrast, pro-angiogenic agents, developed to stimulate blood vessel growth, show a persistent lack of clinical translation. Indeed, despite high hopes and numerous trials, no effective treatment has yet reached the market consisting of millions of patients suffering from acute or chronic ischemic disease. This may validate the old proverb that destroying things is considerably easier than building them. Beyond opposing entropy, what lies at the heart of the matter is the same challenge that faces regenerative medicine in general that is that the creation of complex biological structures requires multi-dimensional cueing of many cell types (2). Nature grows vessels so well during development and postnatal physiological growth and repair. Yet, here we are, after 50 years of active research to decipher the cellular and molecular mechanisms regulating vasculogenesis, angiogenesis and arteriogenesis, still lacking a firm solution of how to build stable and functional blood vessels in human patients. Although bioengineered vascular grafts are making considerable headway toward surgical macrovascular replacement or angioplasty (3), the finer vascular structures, including resistance arteries and arterioles, venules and capillaries require *in situ* instructed growth to functionally integrate tissues. This is the playfield of therapeutic angiogenesis and arteriogenesis. While success stories abound in experimental ischemic models, including in rabbits and pigs, the same treatments have not yielded the expected functional benefits in patients, provoking reevaluation of therapeutic approaches. Notably, we scientists share the blame for this translational failure in that we largely continue to model ischemic diseases experimentally in young animals lacking comorbidities, while our target patients more often than not are old, insulin-resistant or diabetic, hyperlipidemic, overweight and perpetuating poor lifestyle choices (physical inactivity, smoking, high-cholesterol diet…). These factors combine in patients to cause vascular endothelial dysfunction, a well-known marker for elevated cardiovascular disease risk (4).

## The impact of vascular endothelial dysfunction on angiogenic responses

Severely dysfunctional endothelial cells, suffering from altered metabolism (5), high oxidative stress levels (6, 7) and activation of pro-inflammatory pathways (8, 9, 10), are poor responders to angiogenic stimuli. This endothelial resistance has been linked to specific alterations in molecular pathways regulating angiogenesis, such as reduced vascular endothelial growth factor receptor (VEGFR)-2/Flk-1 expression in diabetic patients (11, 12), reduced angiogenic growth factor co-receptor (Nrp1, Syndecans) levels in severely obese, hyperlipidemic mice (13), reduced HIF-1α signaling in endothelium of diabetic mice (14) or altered VEGF downstream signaling linked to activation of protein phosphatases in hyperlipidemic mice (15, 16), but also potentially upregulation of endogenous angiogenesis inhibitors, as reported in diabetic pigs (17). Metabolic alterations of endothelial cells in response to diabetes or aging may also *per se* contribute to poor angiogenic responses as metabolic dysregulation increases Notch signaling (18). With the development of personalized medicine, the screening of patients for tumor mutations is being established in several cancer types as a promising approach to better tailor antitumor and antiangiogenic therapies for each patient (19). Similarly, it may be envisaged that *ex vivo* screening of patient-specific angiogenic resistance mechanisms, for instance, in patient-derived tissue-on-a-chip solutions, could be exploited to guide the selection of specific growth factor cocktails to improve neovascularization responses in ischemic diseases.

Promisingly, some experimental studies attempting angiogenic therapy in old and/or diabetic and/or hyperlipidemic animals with ischemic injury have demonstrated that it is possible to therapeutically induce blood vessel growth and remodeling also under these challenges (20, 21, 22, 23, 24). However, the functional gain to the ischemic territory may remain unsatisfactory despite increased vascularity, notably in human patients suffering from persistent vascular endothelial dysfunction, as demonstrated by impaired flow-mediated vasodilation (FMD). Thus, it seems likely that, similar to the requirement of most antiangiogenic agents to be used in combination with chemotherapy or other tumor cell-targeting drugs in cancer patients, pro-angiogenic therapies should be combined with pharmacological treatments that reduce vascular endothelial dysfunction (25). Given the complex clinical setting, it is noteworthy that exceedingly rare are the experimental studies that evaluate angiogenic therapies in combination with standard cardiovascular drugs. In contrast, in clinical trials conducted with pro-angiogenic agents (gene, protein or cell therapy), the patients are maintained on these various cardiovascular pharmacological treatments. The observation that little progress has been made to stably improve tissue perfusion with angiogenic therapy in patients may suggest that (a) the pharmacological treatments are inefficient to restore endothelial function with persistent angiogenic resistance mechanisms preventing successful revascularization and/or (b) angiogenic agents have been sub-optimally formulated.

Considering the first option, that is whether the issue is endothelial angiogenic resistance, it is currently unknown how the penetrance of endothelial dysfunction may vary between different micro- and macrovascular beds, and further to what degree different pharmacological treatments restore vascular endothelial function in distinct vascular beds. Moreover, it should be considered that endothelial cells are not the only vascular cell type suffering in the presence of cardiovascular risk factors. It is likely that pericyte dysfunction also contributes to vascular malfunction, notably maintenance of vascular barrier, with increased vascular permeability reported in diabetes, for example, in dermal capillaries in humans (26) and in bone marrow capillaries in mice (27). Consideration of such multicellular vascular dysfunctions is expected to benefit future investigations of angiogenic therapy. In the next section, we will shift our focus to the second option, that is how angiogenic therapy may be improved. We will briefly present the evolving strategies applied for therapeutic revascularization, with examples drawn from the ischemic heart, followed by some suggestions for future directions in the field.

## Evolving approaches for therapeutic angiogenesis

### Gene and protein therapies

The first clinical trials on therapeutic angiogenesis were conducted with naked growth factors or plasmid-based gene therapy delivered systemically or intracoronary in patients with acute refractory angina due to non-operable, coronary artery disease (CAD) or following myocardial infarction (MI). These trials, similar to the promising experimental studies that preceded them, were based on treatment with single growth factors: VEGF family members VEGF-A or VEGF-C, or fibroblast growth factor (FGF) family members FGF-1, FGF-2 or FGF-4 (28, 29). Following the translational failures of these initial angiogenic gene and protein therapies, different solutions were proposed for how to improve angiogenic therapy for the clinic (28, 30, 31, 32, 33). These guidelines can be summarized as *increasing the duration of therapy* and/or applying *combinations of growth factors or angiogenic master transcription factors* stimulating the growth of both endothelial and mural cells to achieve arteriogenesis and not only angiogenesis for the generation of functional blood vessels that significantly and sustainably increase tissue perfusion. Indeed, both plasmid and adenoviral gene delivery only lead to short-term (5-10 days) overexpression, as the vectors and infected cells are rapidly cleared by immune cells. However, it remains uncertain what duration of stimulation with growth factors is necessary to create stable and functional blood vessels in patients. Further, while insufficient duration of therapy will fail to induce durable results, unreglated VEGF-A expression levels have been found to induce non-functional angioma-like blood vessel growth (34). The improved approaches that have since been developed include (a) intramyocardial injection of adeno-associated virus (AAV) or lentiviral vectors that significantly prolong gene expression over plasmid or adenoviral vectors (29) and (b) implantable or injectable biopolymers that allow spatiotemporally targeted delivery of multiple growth factor proteins (35).

**Figure 1:**
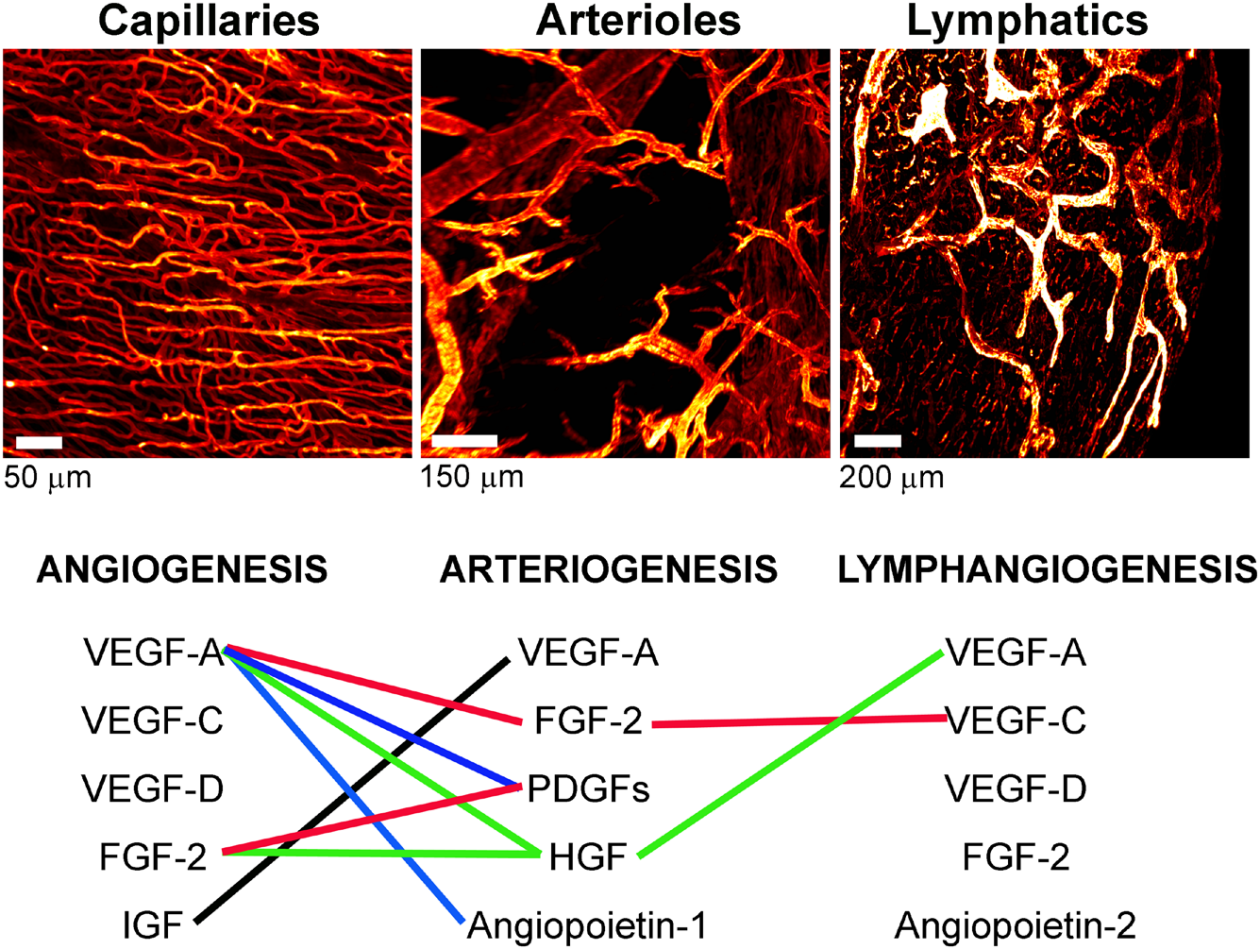
Combinatorial vascular approaches. *Upper panel*: Intermingled vascular networks including capillaries (podocalyxin^+^), arteries and arterioles (α-smooth muscle actin^+^) and lymphatics (LYVE1^+^) in healthy rodent hearts visualized by light sheet imaging of whole-mount stained clarified samples. *Lower panel*: Examples of additive or synergistic growth factor combinations (indicated by solid lines) evaluated for therapeutic angiogenesis, arteriogenesis or lymphangiogenesis using gene or protein therapy (20, 23, 24, 37, 38, 39, 60, 61).

The validity of the latter combinatorial approach has been suggested by experimental reports of durably improved tissue perfusion and function with either bicistronic dual gene delivery (36, 37) or biopolymeric dual protein delivery of multiple growth factors (Fig. 1), for example VEGF-A or FGF-2 combined with either platelet derived growth factor (PDGF)-BB (38, 39) or hepatocyte growth factor (HGF) (40). Promisingly, we and others have shown that such combinatorial growth factor treatments allowed prevention of heart failure development in rats (40) and pigs (41). Of note, therapeutic angiogenesis approaches that have shown benefit in models of experimental ischemia in animals suffering from comorbidities have been based on such combinatorial treatments (20, 22, 23, 24, 42), indicating that this direction may indeed be the way to go to successfully bridge the translational bench-to-bed gap. However, in patients with refractory angina, intramyocardial dual delivery of VEGF-A and FGF-2 failed to increase perfusion, likely due to the use of plasmids rather than viral vectors for delivery (43). This highlights the additional requirement for control over both doses and duration of treatment for efficient induction of vascular growth and remodeling. Future tunable gene or biopolymeric protein therapies, building on these improved approaches, may hold significant promise for clinical success.

### Cell therapies and exosomes

In parallel to these advances in angiogenic gene and protein therapies, cell therapy emerged around the year 2000 as an interesting alternative that would potentially solve both the problem of *duration*, would the cells be stably engrafted, and of delivery of *multiple factors*, as the cellular secretome contains an elaborate cocktail of angiogenic mediators. Initially, the proposed therapy was based on elusive endothelial progenitor cells (EPCs), with the intent to stimulate vasculogenesis (44). Subsequently, therapies were refocused on expansion, priming and delivery of paracrine-acting angiogenesis-promoting cells (45), in the form of either circulating mononuclear cells (CAC) or stem cells of mesenchymal (MSC), bone marrow (BMC) or cardiac (CSC) origin. Currently, in response to poor cell engraftment observed with cell therapy, the field is turning toward the use of cell-free alternatives in the form of exosomes or microvesicles as a shelfable source of regenerative benefit including, but not limited to, stimulation of angiogenesis. These extracellular vesicles (EVs), shedded by cardiac stem cells or by embryonic or induced pluripotent stem cell (iPS)-derived cardiomyocytes, act, following endocytosis, to release intracellularly various angiogenic regulators including transcription factors, growth factors, bioactive lipids, as well as epigenetic regulators such as microRNA (miR) and long-noncoding RNA that together may better orchestrate tissue repair and regeneration (46, 47, 48, 49). The first phase I clinical trial with exosomes for ischemia, aiming at stimulation of angiogenesis as well as modulation of inflammation, is set to start in 2019 in patients with ischemic stroke (clinicaltrials.gov/NCT03384433).

A major weakness of EV-based treatments is that the *duration* and *cell-targeting* of the therapy is currently no better than with liposome-based plasmid gene delivery. Further, the *molecular content* of exosomes remains uncontrolled and heterogeneous with considerable uncertainty regarding cell-dependent impact of exosome-enriched miRs, such as miR146a (50, 51). Finally, it is currently unclear if a single injection of exosomes will suffice to grow stable and functional blood vessels in large animals (52), or if repeated intramyocardial injections will be required. In view of these various limitations, alternative cell-free, synthetic targeted delivery solutions such as polymeric artificial cells may favorably be considered in the future (53).

## Future perspectives: supplementing angiogenesis with lymphangiogenesis?

In most vascularized tissues, blood vessels are accompanied by lymphatic vessels that maintain tissue homeostasis, including regulation of fluid balance and immune cell trafficking (54). We and others have recently shown that cardiac lymphatic structure and function are severely altered following MI, and that poor lymphatic repair (lymphangiogenesis) contributes to adverse cardiac remodeling and dysfunction in rodents (55, 56). To restore organ function in ischemic diseases, stimulation of both vascular systems would thus seem critical to restore tissue perfusion as well as lymphatic drainage to limit ischemia, edema and inflammation and create a favorable microenvironment permissive for tissue repair and regeneration (57). Although experimental data are still lacking on the potential functional benefit of stimulating lymphangiogenesis concomitantly with angiogenesis in ischemic diseases, the first such dual-targeted clinical trial has recently shown safety in patients with CAD (58). Rather than applying growth factor combinations, this trial takes advantage of the dual activities of a member of the VEGF family, VEGF-D, which like VEGF-C stimulates both blood and lymphatic endothelial cell growth (59). Future Phase II investigations with this intramyocardial adenoviral gene therapy approach will reveal whether short-term exposure to a single pleiotropic growth factor will suffice to grow stable and functional blood and lymphatic vasculatures in ischemic hearts, or whether long-term exposure to growth factor combinations again may be required.

## Conclusions

**Figure 2:**
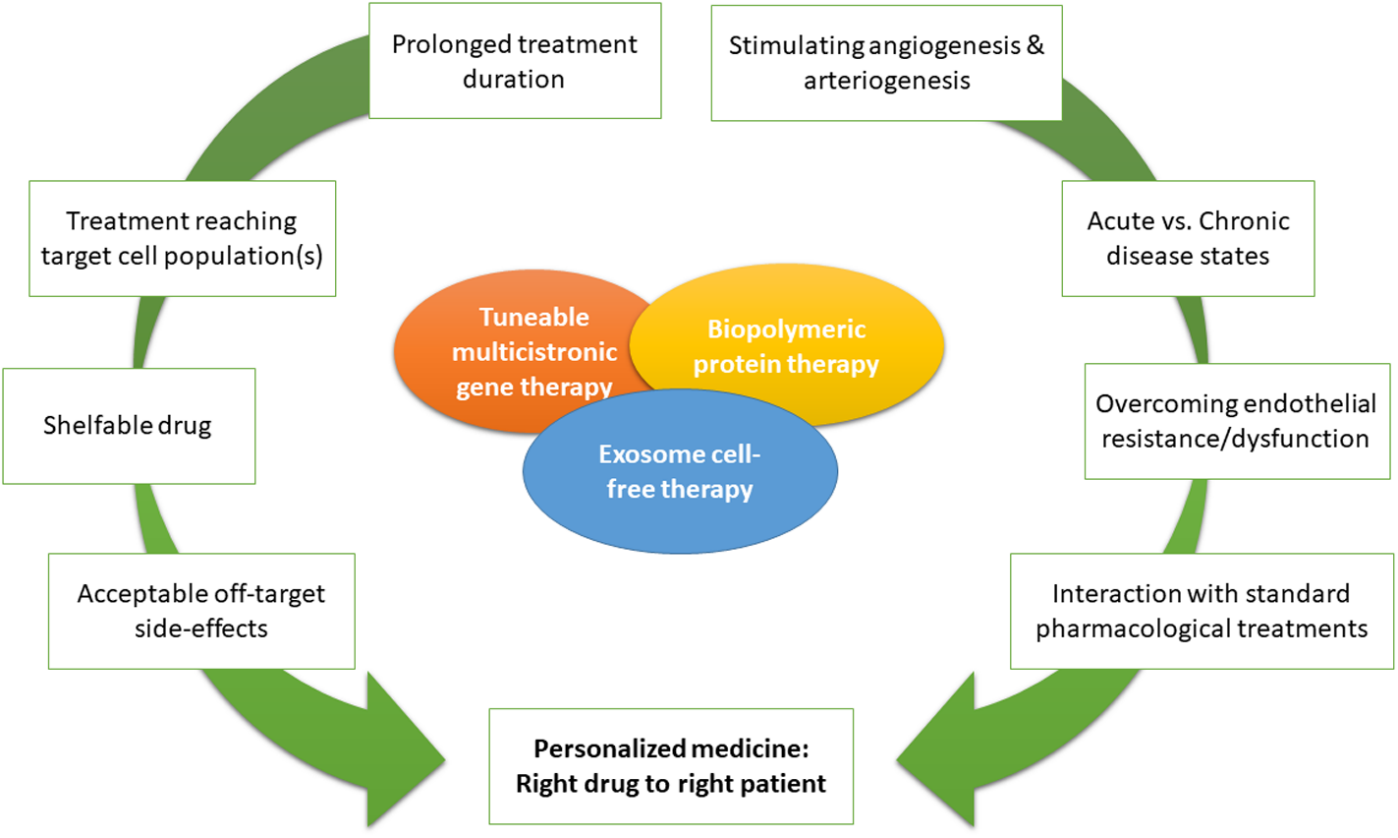
Challenges for therapeutic angiogenesis. In order to grow stable and functional blood vessels in patients with ischemic diseases, several important therapeutic challenges remain, including both basic science and clinical questions (*What cocktail of angiogenic factors must be applied to stimulate both capillary and arteriolar growth? How can therapies overcome potential inherent angiogenic resistance in patients*?) as well as technical hurdles (*How to control spatiotemporally the release of multiple angiogenic factors*?). This may require devising multimodal solutions, based on combinations of different aspects of gene, protein and cell/cell-free exosome therapy, for efficient and safe targeting of angiogenic therapies in patients.

The evolving approaches for therapeutic angiogenesis/arteriogenesis are incorporating hard lessons learned from failing clinical trials, including the need for multiple factors and spatiotemporally-controlled delivery. Whether therapeutic efficacy in patients will come from multicistronic gene therapy, biopolymeric or exosome-based protein/miR/lipid delivery, or combinations of these modalities incorporated into an injectable tissue engineering solution that stimulates both angiogenesis and lymphangiogenesis, remains to be determined. In parallel to these biodrug developments, a more widespread use of pertinent experimental models, mimicking the clinical situation with multiple comorbidities, potentially coupled with patient-derived tissue-on-a-chip *ex vivo* models, is expected to improve clinical translation. Finally, deepened knowledge of patient- and vascular bed-specific vascular endothelial angiogenesis-resistance mechanisms, as well as pharmacological options that reverse them, may be required before angiogenic therapies at last will assume their long-anticipated place as a valid clinical treatment option for ischemic diseases (Fig. 2).

## Declaration of interest

The authors declare that there is no conflict of interest that could be perceived as prejudicing the impartiality of this review.

## Funding

This work was supported by the ERA-CVD (LYMIT-DIS project, E B), FHU REMOD-VHF (Inserm U1096 laboratory, V R), and generalized institutional funds from French Inserm and the Normandy Region together with the European Union: ‘*Europe gets involved in Normandie*’ with European Regional Development Fundhttp://dx.doi.org/10.13039/501100008530 (ERDF): CPER/FEDER 2015 (DO-IT) and CPER/FEDER 2016 (PACT-CBS).
